# Influence of Municipality-Level Mean Income on Access to Aortic Valve Surgery: A Cross-Sectional Observational Study under Japan's Universal Health-Care Coverage

**DOI:** 10.1371/journal.pone.0111071

**Published:** 2014-10-31

**Authors:** Seitetsu L. Lee, Hideki Hashimoto, Takahide Kohro, Hiromasa Horiguchi, Daisuke Koide, Issei Komuro, Kiyohide Fushimi, Tsutomu Yamazaki, Hideo Yasunaga

**Affiliations:** 1 Department of Cardiovascular Medicine, The University of Tokyo, Tokyo, Japan; 2 Department of Health and Social Behavior, The University of Tokyo, Tokyo, Japan; 3 Clinical Research Support Center, The University of Tokyo, Tokyo, Japan; 4 Department of Clinical Epidemiology and Systems, The University of Tokyo, Tokyo, Japan; 5 Department of Clinical Epidemiology and Health Economics, The University of Tokyo, Tokyo, Japan; 6 Department of Clinical Data Management and Research, Clinical Research Center, National Hospital Organization Headquarters, Tokyo, Japan; 7 Department of Clinical Informatics, Jichi Medical University, Tochigi, Japan; 8 Department of Health Policy and Informatics, Tokyo Medical and Dental University, Tokyo, Japan; Hamamatsu University School of Medicine, Japan

## Abstract

**Background:**

Universal health-care coverage has attracted the interest of policy makers as a way of achieving health equity. However, previous reports have shown that despite universal coverage, socioeconomic disparity persists in access to high-tech invasive care, such as cardiac treatment. In this study, we aimed to investigate the association between socioeconomic status and care of aortic stenosis in the context of Japan's health-care system, which is mainly publicly funded.

**Methods:**

We chose aortic stenosis in older people as a target because such patients are likely to be affected by socioeconomic disparity. Using a large Japanese claim-based inpatient database, we identified 12,893 isolated aortic stenosis patients aged over 65 years who were hospitalized between July 2010 and March 2012. Municipality socioeconomic status was represented by the mean household income of the patients' residential municipality, categorized into quartiles. The likelihood of undergoing aortic valve surgery and in-hospital mortality was regressed against socioeconomic status level with adjustments for hospital volume, regional number of cardiac surgeons per 1 million population, and patients' clinical status.

**Results:**

We found no significant differences between the highest and lowest quartile groups in surgical indication (odds ratio, 0.84; 95% confidence interval, 0.69–1.03) or in-hospital mortality (1.00; 0.68–1.48). Hospital volume was significantly associated with lower postoperative mortality (odds ratio of the highest volume tertile to the lowest, 0.49; 0.34–0.71).

**Conclusions:**

Under Japan's current universal health-care coverage, municipality socioeconomic status did not appear to have a systematic relationship with either treatment decision for surgical intervention or postoperative survival following aortic valve surgery among older patients. Our results imply that universal health-care coverage with high publicly funded coverage offers equal access to high-tech cardiovascular care.

## Introduction

Equal access to health care is a major policy concern for achieving health equity in different countries. Universal health coverage (UHC) by means of public funding has been used as a solution. The Medicare program in the United Sates offers near UHC for patients aged 65 years and above. Studies in that country have shown the health benefits and effectiveness in closing the gap across socioeconomic conditions through public health insurance coverage [1], [2]. However, investigations in Canada [3], [4], Australia [5], and Taiwan [6], where UHC has been in effect for decades, have indicated a persisting access gap to high-quality medical care, such as cardiac interventions, which suggests the need for further action to achieve access equity under UHC.

The “World Health Report 2010” of the World Health Organization [7] proposed that to achieve equal access to health care, UHC needs to strike a balance in the proportion of the population covered, the range of available services, and the proportion of direct costs covered by public funding. The three countries mentioned above provide universal coverage in terms of population and service coverage. However, according to the latest health statistics from the Organisation for Economic Co-operation and Development (OECD) and other data sources, the proportion of health-care spending covered by public funding in those places was lower (Canada, 66.4% in 2010 [8]; Australia, 64.4% in 2010 [8]; Taiwan, 53% in 2005 [9]) than in other OECD countries, such as Japan (81.4%) [8]. Given this background, we hypothesized that high coverage by public funding would make a difference in the level of equal access to high-tech invasive care under a UHC scheme.

To examine this issue, we chose to study surgical treatment for aortic stenosis (AS) among an older population. We did so for several reasons. First, older people are more financially vulnerable owing to limited income sources, and their income gap is greater than that among young people [9]. Second, cardiovascular diseases are the leading cause of death globally. An estimated 17.3 million people died through cardiovascular diseases in 2008, representing 30% of all global deaths, according to a report by the World Health Organization [10]. Moreover, AS is a prevalent cardiac condition among older people, and its impact among cardiovascular diseases has increased owing to the growth of Japan's aged population. A study in the United Kingdom reported that aortic valve surgery has increased remarkably in volume owing to the marked rise in the number of older patients suffering from AS [11]. In Japan, where the total population is 127.1 million (10th largest in the world), people aged 65 and above account for over 23% of that population, and the volume of aortic valve surgery exceeds that of coronary artery bypass graft surgery [12]. Third, treatment for AS mostly has an elective status, which makes the treatment decision more discretionary than with emergency conditions, such as acute coronary syndrome [5]. Thus, if an access gap exists, it is likely that surgical treatment for AS among older people will reveal it. We aimed to investigate whether Japan's health-care strategy is associated with smaller access gap for cardiac intervention in an older population.

## Materials and Methods

### Data Source and Patient Inclusion

We used a large claim-bill-based database called the Diagnosis Procedure Combination Database [13] to identify patients aged 65 years old and over who were hospitalized for AS between July 2010 and March 2012. The DPC is a Japanese case-mix classification system linked to a per diem inclusive payment scheme for acute-care hospitals under the national health insurance scheme. The database contains administrative claims and discharge data, which are collected with a standardized electronic format nationwide by the Ministry of Health Labour and Welfare for reimbursement purposes. All 82 university teaching hospitals are obliged to adopt the DPC system, but adoption by community hospitals is voluntary. In 2012, the database included all 82 academic hospitals in Japan and about 1,000 voluntarily participating community hospitals, which covered about 50% of acute-care beds in the country.

The data included a unique identifier of hospitals, patients' residential zip code, comorbidity diagnoses on admission coded using the International Classification of Diseases, Tenth Revision (ICD10) codes, surgical procedures coded using the Japanese claims classification (K-code), and clinical data, such as the New York Heart Association (NYHA) functional classification, smoking index, and body mass index. We identified patients with AS (ICD10 code I350) and AS and regurgitation (I352).

### Municipality Socioeconomic Status

We used municipality-level socioeconomic status (SES) as a marker of socioeconomic inequality. Following previous studies using a US nationwide representative sample of inpatients [14]–[19], municipality SES was assessed by mean household income in the municipality in which the patient lived. Mean municipal household income was estimated as the total municipal household income divided by the number of households, which was derived from the “Statistical Observations of Municipalities (*Shi*, *Ku*, *Machi*, and *Mura*) 2012” [20], published by the Statistics Bureau, Ministry of Internal Affairs and Communications, Japan. We investigated 1,894 municipalities; the median population of the municipalities was 30,644 people (interquartile range, 9,847–83,167), and the median number of households in each municipality was 10,993 (interquartile range, 3,614–31,569). We adjusted the estimated mean municipality-level income for regional consumer price calibrators to enable a cross-regional comparison. Finally, the estimated mean income was categorized into quartiles for further analysis. The first, second, third, and fourth quartiles of income (mean household income adjusted for regional price index) were ≤2,799.5, 2,799.6–3,253.2, 3,253.3–3,719.1, and ≥3,719.2 (1,000 yen), respectively.

### Study Variables and Outcomes

We obtained data on the patients' demographic and clinical status: age (divided into 65–74 years and ≥75 years old); gender; body mass index (measured on admission, categorized into <18.5, 18.5–29.9, and ≥30 kg/m^2^); urgent admission status; pre-hospital dialysis; prior history of stroke; length of stay after surgery; type of surgery (valve repair, aortic valve replacement, replacement of more than two valves, combined surgery with Maze surgery, coronary artery bypass graft, or surgery of the thoracic aorta); and NYHA classification on admission. We defined hospital volume as the number of aortic valve surgery procedures performed during 2011 at each institution and divided the numbers into tertiles (1–35, 36–68, and 69 or more cases/year). We also calculated the number of cardiac surgeons per 1 million population in the municipality.

### Statistical Analysis

We conducted descriptive analyses on variables by municipality SES quartiles. *p* values were calculated using the chi-square test for all variables except length of stay after surgery. The *p* value for length of stay after surgery was calculated using the Kruskal-Wallis test. For multivariable analysis, we fitted generalized estimating equation (GEE) models because the data involved observations clustered by hospital. We regressed the surgical indication (likelihood of undergoing aortic valve surgery) on SES with adjustment for age, gender, body mass index, urgent admission status, pre-hospital dialysis, prior history of stroke, NYHA classification on admission, hospital volume, and regional factor (the number of cardiac surgeons per 1 million population). We also regressed in-hospital mortality after surgery on municipality SES with adjustment for the same set of covariates as in the GEE model of surgical indication as well as length of stay after surgery and type of surgery. All statistical analyses were performed using IBM SPSS version 19.0 (IBM SPSS, Armonk, NY, USA).

### Ethical concerns

Because of the anonymous nature of the data, the need for informed consent was waived by the Institutional Review Board at The University of Tokyo when the study was approved.

## Results

We identified 12,893 older patients with isolated AS who were hospitalized in 393 hospitals between July 2010 and March 2012. Of these patients, 6,784 underwent valve surgery. Table 1 and 2, show the descriptive statistics by municipality SES quartiles. The patients in higher SES quartiles were more likely to be young, thin, smokers, on dialysis, admitted urgently to higher-volume hospitals, and have a diagnosis of lone stenosis (Table 1). The population in the higher SES quartiles was larger. The median population of each SES stratum was 1,812,575 (interquartile range, 1,362,820–5,485,952), 2,631,671 (interquartile range, 1,442,428–5,079,291), 2,855,045 (interquartile range, 2,000,010–7,207,139), and 7,416,336 (interquartile range, 5,581,968–9,058,094), respectively, for the first, second, third, and fourth quartiles of income. Through concerns over low-income patients living in big cities being misclassified as high SES, we also analyzed the database after excluding the five largest cities in Japan (Tokyo, Yokohama, Osaka, Nagoya, and Sapporo), which together account for about 15% of Japan's total population. However, the results of multivariable regression were the same. Surgical treatment was most frequently observed in the lowest municipality SES quartile (56.2%), followed by the highest (52.9%); it was least frequent among the second quartile group (Table 2).

**Table 1:** Patient and institutional characteristics by municipality-level mean income strata (all patients).

		Municipality-level mean income (lowest to highest)				
n = 12,893		1st	2nd	3rd	4th	*p* value
**Patient characteristics**						
Number of symptomatic AS patients		3,228	3,183	3,172	3,310	
Age	≥75	70.0%	70.2%	65.9%	66.1%	<0.001
Female		58.3%	60.7%	57.7%	58.5%	0.072
Smoker		31.0%	29.4%	32.5%	35.3%	<0.001
Prior history of stroke		3.0%	2.8%	2.6%	2.3%	0.249
Dialysis		13.0%	12.3%	14.7%	15.0%	0.003
Urgent admission		17.4%	20.4%	22.1%	20.8%	<0.001
Indication for hospitalization	ASR	15.1%	12.9%	11.9%	11.2%	<0.001
	AS	84.9%	87.1%	88.1%	88.8%	
Body mass index	18.5–29.9	85.1%	85.5%	84.4%	84.1%	0.002
	<18.5	10.2%	11.2%	11.7%	12.5%	
	≥30	4.7%	3.2%	3.9%	3.4%	
NYHA class	0–2	40.8%	39.9%	37.5%	39.5%	0.146
	3–4	20.1%	20.0%	20.5%	19.4%	
	data NA	39.1%	40.1%	42.0%	41.1%	
**Institutional characteristics**						
Hospital volume	1–35	41.8%	39.3%	37.5%	39.8%	<0.001
	36–68	33.5%	32.5%	32.7%	27.3%	
	≥69	24.8%	28.2%	29.9%	33.0%	
Number of patients undergoing surgery		1,814	1,569	1,651	1,750	<.001
(% to total patients)		56.2%	49.3%	52.0%	52.9%	

1st, 2nd, 3rd, and 4th quartiles of income (mean household income adjusted for regional price index): ≤2,799.5, 2,799.6–3,253.2, 3,253.3–3,719.1, ≥3,719.2 (1,000 yen); Hospital volume: the number of aortic valve surgery procedures performed during 2011 at each institution; p values were calculated using the chi-square test.

ASR, aortic stenosis and regurgitation; AS, aortic stenosis, BMI, body mass index; NYHA, New York Heart Association; NA, not available.

**Table 2:** Process and outcome of surgical patients by municipality-level mean income strata.

		Municipality-level mean income (lowest to highest)				
		1st	2nd	3rd	4th	*p* value
Combined surgery	CABG	20.4%	22.2%	22.2%	22.4%	0.436
	TAA	1.2%	0.8%	1.0%	1.1%	0.722
	Maze	6.5%	6.9%	6.5%	8.9%	0.018
Type of surgery	Plasty	0.7%	1.0%	2.4%	5.3%	<0.001
	AVR	91.8%	93.7%	89.1%	85.6%	
	DVR	7.6%	5.4%	8.5%	9.1%	
Length of stay after surgery		29 [22–41]	28[22–41]	27[21–39]	26[19–37]	<0.001*
Postoperative in-hospital death		4.10%	4.30%	4.40%	4.40%	0.973

p values were calculated using the chi-square test and *Kruskal-Wallis test.1st, 2nd, 3rd, and 4th quartiles of income (mean household income adjusted for regional price index): ≤2,799.5, 2,799.6–3,253.2, 3,253.3–3,719.1, ≥3,719.2 (1,000 yen); Length of stay is expressed as mean values [interquartile range].

CABG, coronary artery bypass graft; TAA, thoracic aortic aneurysm; AVR, aortic valve replacement; DVR, dual valve replacement.

Among surgical patients, as shown in Table 2, the type of combined surgery was not significantly different across municipality SES groups—except for Maze operations, which were most frequent in the highest SES group (8.9%). Aortic valve plasty was most frequently performed in the highest SES group, whereas lone aortic valve replacement was commonest in the lowest SES group. Crude in-hospital mortality was 4.1% in the lowest quartile, 4.3% in the second, 4.4% in the third, and 4.4% in the highest quartile (*p* = 0.973). Age-adjusted in-hospital mortality was 4.1, 4.3, 4.4, and 4.4%, respectively, for the first, second, third, and fourth quartiles of income.

Table 3 shows the results of multivariate logistic regression on surgical indication. The second- and third-lowest municipality SES groups were significantly less likely to undergo surgery than the lowest group. Otherwise, there were no significant disparities across municipality SES groups for surgical indication. Patients with severe NYHA scores, those on dialysis, and those admitted to higher-volume hospitals were more likely to receive surgery, whereas patients aged over 75, admitted urgently, and with low body mass index were less likely to undergo surgery.

**Table 3:** Multivariate model of GEE for the likelihood of undergoing valve surgery.

		Unadjusted OR	95% CI			Adjusted OR	95% CI		
Municipality-level mean income	4	0.87	0.70	-	1.09	0.84	0.69	-	1.03
	3	0.85	0.69	-	1.03	0.80	0.66	-	0.97
	2	0.76	0.63	-	0.91	0.72	0.60	-	0.86
	1	1				1			
Hospital volume	≥69	3.78	2.96	-	4.82	3.36	2.67	-	4.24
	36–68	3.39	2.81	-	4.08	3.24	2.68	-	3.91
	1–35	1				1			
Number of cardiac surgeons per 1 million population (per one increment)		0.98	0.89	-	1.08	0.97	0.93	-	1.01
Age	≥75	0.62	0.57	-	0.67	0.74	0.67	-	0.81
Female		0.82	0.77	-	0.88	1.02	0.94	-	1.11
Smoker		1.23	1.12	-	1.34	1.13	1.02	-	1.26
NYHA class	Data NA	1.05	0.91	-	1.21	1.17	1.01	-	1.35
	4	0.72	0.56	-	0.91	1.52	1.22	-	1.91
	3	1.38	1.11	-	1.72	1.88	1.54	-	2.29
	2	1.53	1.27	-	1.84	1.63	1.38	-	1.92
	0–1	1				1			
Prior history of stroke		0.95	0.74	-	1.23	1.09	0.85	-	1.39
Dialysis		1.36	1.21	-	1.53	1.38	1.2	-	1.57
Urgent admission		0.30	0.26	-	0.33	0.34	0.29	-	0.38
BMI	≥30	1.02	0.85	-	1.22	0.95	0.78	-	1.16
	<18.5	0.74	0.66	-	0.83	0.78	0.69	-	0.88
	18.5–29.9	1				1			

GEE, generalized estimating equation; Municipality-level mean income represents mean household income adjusted for regional price index. 1st 2nd, 3rd, and 4th quartiles of income: ≤2,799.5, 2,799.6–3,253.2, 3,253.3–3,719.1, ≥3,719.2 (1,000 yen); Hospital volume: the number of aortic valve surgery procedures performed during 2011 at each institution.

AS, aortic stenosis; BMI, body mass index; CI, confidence interval; NYHA: New York Heart Association; NA, not available; OR, odds ratio.

Table 4 presents the results on postoperative in-hospital mortality. After adjusting for patient, hospital, and regional factors, municipality SES quartiles exhibited no statistically significant association with mortality. Patients who were older, female, with NYHA class IV, had a stroke history, and were undergoing dialysis had significantly higher mortality, whereas those admitted to high-volume hospitals showed significantly lower in-hospital mortality.

**Table 4:** Multivariate model of GEE for postoperative in-hospital death among surgical patients.

		Unadjusted OR	95% CI			Adjusted OR	95% CI		
Municipality-level mean income	4	1.07	0.76	-	1.49	1.01	0.68	-	1.49
	3	1.07	0.76	-	1.51	1.09	0.73	-	1.64
	2	1.03	0.74	-	1.45	1.15	0.77	-	1.71
	1	1				1			
Hospital volume	≥69	0.58	0.42	-	0.80	0.51	0.35	-	0.73
	36–68	0.66	0.49	-	0.87	0.66	0.47	-	0.92
	1–35	1				1			
Number of cardiac surgeons per one million population (per one increment)		1.05	0.93	-	1.18	1.02	0.89	-	1.17
Age	≥75	1.28	0.99	-	1.67	2.08	1.55	-	2.79
Female		0.88	0.69	-	1.12	1.44	1.02	-	2.03
Smoker		1.14	0.89	-	1.45	1.21	0.89	-	1.63
NYHA class	Data NA	1.99	1.30	-	3.04	1.77	1.11	-	2.81
	4	4.52	2.76	-	7.41	2.76	1.51	-	5.04
	3	1.88	1.16	-	3.03	1.47	0.85	-	2.52
	2	0.81	0.50	-	1.30	0.88	0.53	-	1.47
	0–1	1				1			
Prior history of stroke		1.94	1.11	-	3.37	2.17	1.14	-	4.13
Dialysis		22.06	16.22	-	30.0	23.12	16.34	-	32.7
Urgent admission		2.38	1.78	-	3.18	1.31	0.89	-	1.91
Length of stay after surgery (per one increment)		1.01	1.01	-	1.01	1.00	1.00	-	1.01
BMI	≥30	1.22	0.69	-	2.18	1.28	0.68	-	2.41
	<18.5	2	1.47	-	2.72	1.09	0.76	-	1.56
	18.5–29.9	1				1			
Combined surgery	CABG	1.53	1.18	-	1.98	1.26	0.93	-	1.70
	TAA	2.8	1.31	-	6.00	2.58	1.04	-	6.42
	Maze	1.18	0.75	-	1.86	1.01	0.60	-	1.71
Type of surgery	DVR	0.91	0.51	-	1.62	0.81	0.42	-	1.55
	AVR	0.43	0.26	-	0.69	0.43	0.24	-	0.79
	Plasty	1				1			

GEE, generalized estimating equation; Income represents mean household income adjusted for regional price index. 1st, 2nd, 3rd, and 4th quartiles of income: ≤2,799.5, 2,799.6–3,253.2, 3,253.3–3,719.1, ≥3,719.2 (1,000 yen); Hospital volume: the number of aortic valve surgery procedures performed during 2011 at each institution;

OR, odds ratio; CI, confidence interval; AS, aortic stenosis; BMI, body mass index; NYHA, New York Heart Association; CABG, coronary artery bypass graft; TAA, thoracic aortic aneurysm; AVR, aortic valve replacement; DVR, dual valve replacement; NA, not available.

## Discussion

Using a large claim-based database, our analysis did not detect any systematic disparity across municipality SES groups in the outcome of surgical treatment for older AS patients in Japan. However, the second- and third-lowest SES groups were significantly less likely to receive surgery than the lowest group. The trend was not linear but U-shaped. We have no good explanation for this trend.

Patients with AS have not previously been fully investigated with regard to the relationship between their SES and operative outcome. After controlling for the patient's clinical condition and hospital characteristics, a US study using a nationally representative sample [14] reported that municipality household income was linked to unequal postoperative survival following various surgical procedures. That investigation showed municipality-level income to be an independent factor of in-hospital death after aortic valve replacement. By contrast, numerous studies of patients with ischemic heart disease have identified a socioeconomic disparity in access to and clinical outcomes of cardiovascular interventions [3]–[6], [17], [21]–[23]. Unlike these studies', our results did not suggest socioeconomic disparity in surgical indication and subsequent outcome after aortic valve surgery among older AS patients following hospital referral. We conducted a similar analysis with respect to length of stay and cost per day but found no significant differences among the SES groups (data not shown, available from the authors on request). The results of that analysis lend further support to our finding that the indication decision, resource use, and surgical outcome for AS in older people under Japan's UHC coverage do not depend on the local socioeconomic conditions of a patient's residential area.

A possible explanation for our results may relate to the uniqueness of Japan's UHC: the proportion of public funding covering total health expenditure is high [8]. High coverage by public funding is mainly achieved in two ways. One is the reduced copayment for people aged over 75, which has been shown to have a positive health impact among older people [24]. The other is public subsidies to prevent catastrophic payment, whereby out-of-pocket payments that exceed a monthly limit according to annual household income levels are reimbursed [8]. TRICARE is a UHC in the United States with almost full governmental coverage for all active-duty and retired military service personnel as well as their dependents. Previous reports studying the population under TRICARE [25], [26] have shown that even in the United States with a wide range of SES, UHC with high publicly funded coverage offered equal access to high-tech care. We suppose that this policy of keeping out-of-pocket payments affordable for a patient's household plays an important role in a patient choosing advanced care, such as cardiovascular interventions. However, there is still a lack of studies examining access to and quality of health care under systems that offer high public coverage. Further investigations are warranted especially in countries with UHC and high publicly funded coverage, such as Germany, Norway, and the Netherlands.

In terms of supply-side control, Japan's UHC is unusual in that its fees are uniform across the nation; this may reduce regional disparity in the allocation of facilities that perform cardiovascular interventions. Using a nationwide registry, Nakamura et al. [27] detailed how Japanese hospitals provide high-quality care for patients with acute myocardial ischemia either during regular hospital hours or after hours regardless of location. Their results support our argument that resources for and the outcome of cardiovascular interventions in Japan do not depend on the local socioeconomic conditions of a patient's residential area. The socioeconomic circumstances under Japan's UHC may enable equal resource use after hospital referral regardless of local SES.

In line with repeated findings in other studies, our analysis confirms the significant impact of hospital volume on the propensity for surgical intervention and in-hospital mortality [28], [29]. Since admission to a high-volume hospital was significantly related to higher SES neighborhood, type of surgery, and better surgical outcome, we conducted an additional multivariate analysis that excluded hospital volume: we were concerned that hospital volume may have over-controlled the impact of municipality SES. However, this analysis simply confirmed our former findings. We also checked variance inflation factors for each variable, and all values were less than 2. Hence, we believe that our models were not seriously affected by multicollinearity. We speculate that the observed association between municipality SES and hospital volume may simply reflect the positive effect of treatment in an urban high-volume center.

## Policy Implications

Our results suggest that the UHC may have contributed to equal access to and quality of cardiovascular surgery for AS patients in Japan. Small out-of-pocket payments that receive a high level of coverage by public funding may have enabled patients to undergo medically appropriate treatment regardless of their SES. Full reimbursement under the UHC and uniform fees across the nation may also reduce regional disparities in facilities that perform cardiovascular interventions. The socioeconomic circumstances achieved under Japan's UHC may have enabled equal resource use after hospital referral regardless of municipality SES. Recently, there has been discussion about the weakened financial sustainability of Japan's health-care system and the possible change to a more austere policy in the near future. We suggest that further monitoring will be necessary to examine how such policy changes could impact the relationship between SES and the outcome with high-tech cardiovascular care.

## Strengths and Limitations of This Study

The key strength of our study is that we covered 98.6% of the patients who underwent isolated aortic valve surgery in 2011; we based this figure on the annual report of the Japanese Association for Thoracic Surgery [12]. However, several limitations should be acknowledged. First, we could not obtain information about individual SES, and the aggregated income data we used may have ignored regional income disparity; this could have led to an underestimation of the socioeconomic gradient of a surgical indication and outcome. Further, we established a regional composite SES index based on a previous study [30], which included unemployment rate, dwelling area per household, proportion of households receiving public assistance, percentage of persons with higher education, per capita income, percentage of owned houses, and percentage of aged single households. However, the tendency and results using the regional composite SES index were the same as those when we employed the municipality-level mean income. A review article [31] about SES and health in the Japanese population concluded that income and SES exhibited a significant relationship, and so we adopted municipality-level mean income as a proxy for municipality SES.

Second, the claims data we used in this analysis suffered from a lack of detailed clinical information and possible miscoding of diagnoses, which may have caused our null findings. Third, our model for surgical indication failed to exhibit good model fitness, which indicates that factors influencing the surgical indication may have been incorrectly specified in our model. However, our results are in accordance with a 7-year longitudinal nationwide report from the United States [32]; it showed that differences between ethnic groups in undergoing cardiovascular procedures narrowed markedly once a serious illness developed and there was adequate insurance coverage. Fourth, 30-day mortality would be more robust to confounding by length of stay for assessing postoperative mortality. However, we were unable to use 30-day mortality in our model owing to data limitations (median value of postoperative length of stay in surgical patients, 21 days; interquartile range, 16–30). Instead, we used length of stay after surgery as a covariate of the model. Finally, through lack of proper data, we were unable to consider the effects of SES on waiting time for elective surgery, timing of hospital referral, and mid-term outcome of patients: the DPC database included in-patient data only. Future investigation using a population based on registry data is warranted. Whether the mid-term outcome differs across socioeconomic groups requires further investigation.

## Conclusions

Under the current Japanese health-care system, municipality SES did not appear to influence systematically either the treatment decision for surgical intervention or postoperative survival following aortic valve surgery. Our results imply that a UHC with high coverage by public funding allows equal access to high-tech care. According to the three-dimensional model of UHC, policy makers should carefully design their systems if they aim to achieve equity in health-care access within a population in light of underlying socioeconomic disparities.
